# Stem cell therapies and benefaction of somatic cell nuclear transfer cloning in COVID-19 era

**DOI:** 10.1186/s13287-021-02334-5

**Published:** 2021-05-12

**Authors:** Birbal Singh, Gorakh Mal, Vinod Verma, Ruchi Tiwari, Muhammad Imran Khan, Ranjan K. Mohapatra, Saikat Mitra, Salem A. Alyami, Talha Bin Emran, Kuldeep Dhama, Mohammad Ali Moni

**Affiliations:** 1 ICAR-Indian Veterinary Research Institute Regional Station, Palampur, Himachal Pradesh India; 2 Stem Cell Research Centre, Department of Hematology, Sanjay Gandhi Post-Graduate Institute of Medical Sciences, Lucknow, India; 3 Department of Veterinary Microbiology and Immunology, College of Veterinary Sciences, Uttar Pradesh Pandit Deen Dayal Upadhyaya Pashu Chikitsa Vigyan Vishwavidyalaya Evam Go Anusandhan Sansthan (DUVASU), Mathura, 281001 India; 4 Hefei National Lab for Physical Sciences at the Microscale and the Centers for Biomedical Engineering, University of Science and Technology of China, Hefei, China; 5 Department of Chemistry, Government College of Engineering, Keonjhar, Odisha India; 6 Department of Pharmacy, Faculty of Pharmacy, University of Dhaka, Dhaka, 1000 Bangladesh; 7 Department of Mathematics and Statistics, Imam Mohammad Ibn Saud Islamic University, Riyadh, 11432 Saudi Arabia; 8 Department of Pharmacy, BGC Trust University Bangladesh, Chittagong, 4381 Bangladesh; 9 Division of Pathology, ICAR-Indian Veterinary Research Institute, Izatnagar, Bareilly, Uttar Pradesh 243 122 India; 10 WHO Collaborating Centre on eHealth, UNSW Digital Health, Faculty of Medicine, School of Public Health and Community Medicine, UNSW Sydney, Sydney, NSW 2052 Australia

**Keywords:** SCNT, Genome reprogramming, Stem cells, SARS-CoV-2, COVID-19, Regenerative medicine, Bio-pharming

## Abstract

**Background:**

The global health emergency of COVID-19 has necessitated the development of multiple therapeutic modalities including vaccinations, antivirals, anti-inflammatory, and cytoimmunotherapies, etc. COVID-19 patients suffer from damage to various organs and vascular structures, so they present multiple health crises. Mesenchymal stem cells (MSCs) are of interest to treat acute respiratory distress syndrome (ARDS) caused by SARS-CoV-2 infection.

**Main body:**

Stem cell-based therapies have been verified for prospective benefits in copious preclinical and clinical studies. MSCs confer potential benefits to develop various cell types and organoids for studying virus-human interaction, drug testing, regenerative medicine, and immunomodulatory effects in COVID-19 patients. Apart from paving the ways to augment stem cell research and therapies, somatic cell nuclear transfer (SCNT) holds unique ability for a wide range of health applications such as patient-specific or isogenic cells for regenerative medicine and breeding transgenic animals for biomedical applications. Being a potent cell genome-reprogramming tool, the SCNT has increased prominence of recombinant therapeutics and cellular medicine in the current era of COVID-19. As SCNT is used to generate patient-specific stem cells, it avoids dependence on embryos to obtain stem cells.

**Conclusions:**

The nuclear transfer cloning, being an ideal tool to generate cloned embryos, and the embryonic stem cells will boost drug testing and cellular medicine in COVID-19.

## Highlights

- MSCs serve as potential regenerative medicine and drug-testing tools in COVID-19.
- SCNT cloning is an alternative means of obtaining nuclear-transfer embryonic stem cells (NT-ESCs) which negate dependence on embryos to generate ESCs.
- Nuclear transfer cloning produces cloned transgenic animals for clinical investigations and recombinant therapeutics for health applications.

## Introduction

Coronavirus disease 2019 (COVID-19), as a pandemic, has affected badly several areas of human life. Especially countries with weak economies, limited resources, and poorly developed health system are in big trouble [1–3]. However, the prevalence rates of infection in developed countries like the USA and several other countries are very high [4, 5]. But, these countries have ample healthcare facilities for patients to compensate for their technology and research and provide better protection to their frontline medical practitioners. According to the unpublished report in certain underdeveloped countries, due to the limitation of available resources, a significant number of healthcare workers have lost their lives due to COVID-19. Whatever be the circumstances, everyone is working up to the potential within available resources to fight against COVID-19. Several therapies, like cellular therapies, plasma therapies, antivirals, certain inhibitors, etc., are being applied to treat patients [6]. Plasma therapy is an adjuvant preventive medication for critically sick COVID-19 patients before long-term clinical trial treatment alternatives are available [7]. Plasma from recovering patients, mainly after serious infection, can produce large amounts of polyclonal, pathogen-specific antibodies [8]. These antibodies confer purposeful protective immunity to recipients with similar infection conditions [9]. Convalescent plasma is assumed to perform primarily by counteracting viral components in viral pathogenic diseases [10]. Additionally, some available antiviral agents like remdesivir, favipiravir, ribavirin, etc., have been established with promising activities against SARS-CoV-2 [11].

The rodents (mice, rats, and rabbits); non-human primates (NHPs), mainly the macaques (*Macaca mulatta*), Cynomolgus monkeys (*Macaca fascicularis*), and African green monkey (*Chlorocebus sabaeus*); and some farm animals, such as pigs, cats, and dogs and the large domestic animals [12], are on the leading edge as model species to study pharmacology, immunology, nutrition, and drug testing and comprehend and cure the human diseases. Microbiome-targeted therapies [13–15], phage therapy [16, 17], antibody-antibiotic conjugates, stem cell-based therapies [18, 19], and nanomedicine [20–22] have emerged as prospective unconventional therapies to cure human diseases. Cellular medicine uses living cells, tissues, or biological processes to repair and regenerate the body exclusive of dependence on major surgery. In humans, cell therapy is used to treat and cure diseases such as cancer by injecting living cells into patients. Examples of the diseases treated with stem cells and regenerative medicine include spinal cord injuries (SCI) [23], tendon and ligament injury and musculoskeletal healing [24], diabetes [25, 26], cancers [27–29], and cognitive dysfunctions [30, 31].

Allogenic, autologous, xenogenic, embryonic stem cells (ESCs); MSCs and neural stem cells; and hematopoietic stem cell therapies are expansively used in humans. In animals, stem cells are used to treat tendon, ligament, and cartilage injuries in sports animals [32–34]. Mature or differentiated cell transplantation and de-differentiated cells genetically reprogrammed or induced pluripotent stem cells (iPSCs) are used to treat a range of human diseases and pathologies [18, 35]. Several clinical and preclinical trials have confirmed the potency of stem cell therapy. Also, 88 trials for COVID-19 patient populations were reported as exploring the protection and effectiveness of stem cell transplantation therapy or stem cell-derived exosomes [36]. Investigated symptoms involve COVID-19 with severe pneumonia, respiratory distress, acute respiratory distress syndrome (ARDS), and pulmonary fibrosis. The bulk of trials were recorded for “COVID-19” (19 out of 88) and “severe pneumonia” patients (37 out of 88). As per a meta-analysis of 50,466 hospital-admitted COVID-19 patients, 14.8% of COVID-19 patients developed ARDS [37]. Interestingly, only one of 88 cellular therapy-based trials ascertained the adverse effects, including severe kidney injury. Otherwise, most of them had favorable benefits with stem cell therapy [38].

This review presents the potential of stem cell therapies, as well as the support of SCNT cloning in cellular medicine in the COVID-19 era. The concepts and ideas emanating from this study can be considered to enhance drug testing and discovery for prevention against COVID-19.

## COVID-19 and cellular medicine

The SARS-CoV-2 infections including its variants are new and deadlier than other ssRNA coronaviruses, which are relatively innocuous. Ever since its emergence in mid of December 2019 in Wuhan city, China, SARS-CoV-2 has spread across continents and countries, infecting more than 128 million people while killing nearly 3 million as of March 30, 2021 (COVID-19 Coronavirus Pandemic, World meters, 2021).

It posed high global health concerns due to its ongoing pandemic and crippled the human life socio-economically. The infected people are under enormous stress due to fear of fatal outcomes. SARS-CoV-2 causes multiple health problems [39], such as inflammation, hypoxia, oxidative stress, mitochondrial dysfunction [40], myocarditis due to intense immune response [41], propensity of clotting in micro- and large blood vessels [42], direct infection of cardiomyocytes and cardiogenic shock [43], neurological and neuropsychiatric illnesses [44], kidney damage [45], and immunopathological conditions including cytokine storm [39, 46].

A cytokine storm, also referred to as hypercytokinemia, is a physiological response in people and other living organisms that triggers an unregulated and excessive release of pro-inflammatory signaling molecules called cytokines [47]. Cytokine storm as a hallmark of COVID-19 is characterized by the presence of various immune cells. Elicited immune response by these cells releases interleukin (IL) IL-2, IL-6, IL-7, interferon gamma, tumor necrosis factor, granulocyte colony-stimulating factor, inducible protein-10, and macrophage chemoattractant protein-1, which suggests a well-determined effort by the immune system, but is misconceived [48, 49]. As the MSCs possess regenerative, immunomodulatory, and anti-inflammatory properties stemmed from their stemness, these cells can play an essential role as therapies and patient health management in COVID-19 [50, 51].

COVID-19-associated cytokine storm resulting from displaced immune system activation drives unbalanced immune response, inflammation, and intravascular coagulation and induces vascular damage in patients. These complications result in vascular cardiopulmonary collapse and confer a poor prognosis [52, 53]. Besides these complications, ACE2 receptors are widely distributed in tissues of various organs such as kidneys, cardiac muscles, and smooth and endothelial muscles of multiple organs, which explicates that SARS-CoV-2 can generate systemic diseases and damage various organs [51]. Under these threatening conditions, in addition to an appropriately optimized immune response from the system, repair of damaged endothelial is a crucial question in recovery and getting rid of COVID-19. Given these preliminary scenarios, endothelial progenitor cells (EPCs) may play a key role in inhibiting or reversing this damage in these patients [54]. Current evidence indicates EPCs of bone marrow origin as potential candidates for vascular repair and considerably promising therapeutics [55, 56]. In critical patients and severe cases of COVID-19, EPCs can play an essential role in maintaining vascular endothelial functions, hence contributing to the stem cell therapies in COVID-19 patients [54].

Rapid research advances have paved the ways to develop potential therapeutic interventions, vaccines, drugs, therapies, and immunomodulators to tackle COVID-19, few of which are in the last phases of clinical trials. Success has been achieved to develop anti-SARS-CoV-2 vaccines, and vaccination is currently in progress [57–64]. Immunological interventions, immune-mediated, gene-editing-based therapeutics, and cell-based therapies [65–72] utilizing natural killer (NK) cells [73, 74], T cells [75, 76], convalescent plasma, neutralizing antibodies, monoclonal antibodies [62, 77, 78], cytokines, interferons [79, 80], toll-like receptors (TLRs) [81, 82], and stem cell-based therapies [7, 83] have also been exploited to curb the SARS-CoV-2.

## Stem cell-based COVID-19 therapy

Individual humans’ immune system plays a fundamental role in reclamation and protection against infections. Therefore, evading the detrimental cytokine storm elicited in response to virus invasion is very important in critically affected COVID-19 patients. Immunomodulatory and regenerative properties of MSCs make them promising candidates to cure or lessen the suffering from COVID-19. Intravenously administered MSCs migrate and cumulate in pulmonary microvasculature, where they secrete various paracrine factors which protect and rejuvenate pulmonary epithelial cells and improve lung functioning [84]. In addition, the administered MSCs due to their ability to home and act on injured organs such as the heart, liver, and kidney are useful as cellular medicine [85].

Stem cell-based therapies can be stated as any treatment strategies for severe health issues or medical conditions that implicate the usage of any sort of feasible humans’ stem cells, such as induced pluripotent stem cells (iPSCs), ESCs, and adult stem cells for allogeneic and autologous therapies. Stem cells may confer the potential remedy if tissue and organ transplantation are needed through their capacity to distinguish between the specific cell types used to repair diseased tissues [86, 87]. Currently, stem cell-based therapies are being evaluated against SARS-CoV-2 and for their potential in treating COVID-19 patients [7, 83, 88–90]. MSCs, also recognized as mesenchymal stromal cells or medicinal signaling cells, are multipotent stromal cells that can set apart into a choice of cell sorts, including bone cells (osteoblasts), cartilage cells (chondrocytes), muscle cells (myocytes), and adipocytes which can differentiate into other cell lineages [91–93]. MSCs with their immunomodulatory and regenerative properties have been put forward as promising therapeutic regimens for severe SARS-CoV-2 and can impact the management of COVID-19 patients [94–103]. MSCs can be harvested from different adult tissues with their regenerative and multipotent capacities [104]. Leng et al. investigated the effect of a single dose of transfused MSCs in seven COVID-19 patients and reported a decrease in cytokine levels and patient’s improvement within 2 days. Authors further reported that natural killer and T cells responsible for cytokine storm also disappeared after 6 days with no side effects [105]. O’Driscoll mentioned a case study in Baoshan, China, which also showed a similar outcome [106]. Another case study of MSC infusion showed significant patient improvement after 24 h without considerable side effects [107]. MSCs communicate via paracrine mechanisms such as excretion of extracellular vesicles (EVs), including exosomes and macrovesicles [108–110]. The EVs (exosomes and ectosomes) affect immune cells by induction of anti-inflammatory macrophages, regulatory dendritic cells, regulatory T and B cells, and inactivation of T cells; halt cytokine storm and pro-angiogenic action; and secrete a variety of bioactive and immunomodulatory factors. These properties make them suitable for treatments of severe cases of COVID-19 [83, 111–119]. Published reports suggest that many benefits of MSCs can be achieved by exosomes and ectosomes while avoiding cellular transfusion and associated retaliation [120–122]. So, MSC-derived EVs of various sources like the umbilical cord, peripheral blood, amniotic fluid, etc., can also give almost all MSC therapeutic benefits and are being evaluated for the same [106, 123]. Promising therapeutic aspects of EVs of MSCs include nasal administration or inhalation and non-replicative nature that are void of side effects like uncontrolled cell division associated with cell therapy [106]. Otherwise, MSCs inhibit tissue fibrosis and cell death, releasing immunomodulatory factors that suppress the cytokine storms in COVID-19 patients [118]. Intravenous infusion of perinatal, i.e., umbilical cord and Wharton’s jelly, MSCs improved pulmonary functions and symptoms in COVID-19 pneumonia. The treated patients had improved CD3^+^, CD4^+^, and CD8^+^ cell count and decreased IL-6, TNF-α, and C-reactive proteins [124].

Several trials have been conducted to evaluate the clinical avenues and therapeutic potentials of MSCs in treating COVID-19 patients. Few of them have proved the safety and efficacy of MSCs, suggesting their potent clinical applications and prospects to fight against COVID-19 [86, 94, 98, 101, 125–136]. Thus, MSCs might constitute potentially life-saving treatment options for critically ill COVID-19 patients, could reduce severity and longevity of ARDS in such patients, and reduce the associated mortality rates during the pandemic. However, in-depth studies and large-scale trials for confirming and validating the safety, potency, and efficacy of MSCs, supported with reliable and sufficient scientific evidence, would aid in optimizing the use of MSCs and MSC-derived products, cell-free therapies, and stem cell transplantation therapy to alleviate and treat critically severe COVID-19 pneumonia and illness. Explorative research is warranted to resolve prominent challenges such as the fate of administered MSCs, validating their safety and efficacy, homing capacity, resistance to the disease microenvironment, and stem cell quality management. Also, due attention is needed for adopting strict ethical, regulatory permissions and clinical guidelines for developing safer and effective MSC-based therapies for patients with COVID-19 [128, 137–140].

Two clinical studies were undertaken to consider the potential improvements of MSCs in COVID-19 patients with high fever, dyspnea (difficulty in breathing), and pneumonia. A total of 10 individuals were selected as subjects, and the analysis was performed on seven SARS-CoV-2-positive patients, four of whom had severe symptoms, two exhibiting common sorts of the syndrome, and one of whom was seriously ill. The remaining three patients were recruited in placebo monitoring for acute symptoms. Clinical-grade human MSCs were injected intravenously into all seven patients. The patients were administered with 1 × 10^6^ MSCs per kilogram of body weight while their situation was seriously deteriorating and studied for a cumulative duration of 14 days. Before the administration of MSCs, patients were recorded with elevated fever (body temperatures varying from 38.5 to 39 °C), lowered oxygen saturation, dyspnea, and pneumonia. This study ascertained that the symptoms, which had appeared in patients before infusions, receded within 2–4 days after administrating the MSCs. The oxygen saturation at rest, with or without oxygen uptake, increased to around 95% [50, 141].

Intravenously administered MSCs were found to travel straight into the lungs where several factors secreted by MSCs played a major role in immunomodulation, protecting alveolar epithelial cells, combating pulmonary fibrosis, along with providing improved lung functions, which make greater benefits for treating severe pulmonary conditions in COVID-19 [124]. Another research conducted by Liang et al. [142] included a severe ventilator-ridden COVID-19 patient treated with a human umbilical cord MSC (hUCMSC). The patient was monitored at intervals every 3 days with three infusions of 5 × 10^7^ hUCMSC and was capable of walking only 4 days after the second MSC infusion. The patient did not display detectable side effects, and the critical parameters such as T-cell counts were returned to the normal range [142]. Another study was carried out to observe the efficacy of intravenous infusion of bone marrow-derived MSCs in ARDS [143]. The research was undertaken in the first step with the intravenous injection of stromal MSCs in 9 ARDS patients. The MSC infusion was found to be effective and showed no adverse effects [143]. Phase 2 of the study indicated that a sheep model with bacterial pneumonia was less severely impaired by acute lung damage in therapy with hMSCs [144].

Following an intravenous infusion, significant populations of the MSCs were found to migrate and gather in the lungs where they protected alveolar epithelial cells, re-established pulmonary microenvironment, and cured lung dysfunction besides preventing the pulmonary fibrosis [145]. Systemic infusion of 5 × 10^7^ human umbilical cord MSCs (UC-MSCs) through intravenous injection every 3 days for 3 three times improved health outcomes in the patients and hence could be considered as a prospective alternate therapy to treat COVID-19 in general and elderly patients [146].

The patients having acute SARS-CoV-2-induced ARDS accompanied by severe hypoxemia were given intravenous infusions of 200 × 10^6^ UC-MSCs daily (6 cases) or placental MSCs (5 cases) [147]. The treatment did not cause any adverse effects after 24–48 h post cell infusion. The study concludes that multiple infusions of high doses of allogenic UC- and placental MSCs can swiftly improve COVID-19-induced ARDS in some patients. However, MSC therapy should be avoided in exceptional patients who are prone to develop sepsis or multi-organ failure [147]. Nevertheless, studies emphasize on the need for randomized multicenter clinical trials with long-term follow-up of the stem cell treatments [83, 147, 148]. Indeed, the use of MSCs against COVID-19 is still at the initial stages. As stem cell treatments have already revealed positive outcomes in patients with pulmonary pathologies, there are prospects of hopeful therapies using assorted types of MSCs in immunomodulation, regenerative medicine, cell or tissue engineering, and anti-inflammatory treatments [89].

A parallel assigned controlled, non-randomized, phase 1 clinical trial using intravenous infusion of 3 × 10^7^ cells per fusion on days 0, 3, and 6 was conducted to evaluate the safety and efficacy of UC-MSCs to treat severe COVID-19 pulmonary pathology [148]. Despite diminutive initial side effects viz., transient facial flushing, slight fever, and transient hypoxia at 12 h post-infusion, the patients recovered soon and were discharged from the clinics. The study infers that intravenous UC-MSC infusion is safe and tolerable in moderate and severe COVID-19 patients [148]. UC-MSCs have been suggested for compassionate applications in critically ill COVID-19 patients to decrease mortality and morbidities [83]. In countries like China where modalities to prevent COVID-19 were limited, the patients were treated with stem cells such as UC-MSCs [83].

Indeed, MSCs are very useful in the treatment of COVID-19 for the reason that they mitigate or decrease the cytokine storm through their immunomodulatory capacity via inhibition of T and B cell proliferation and through effective regulation of pro-inflammatory cytokines to improve the microenvironment for endogenous repair [149, 150]. With regard to COVID-19, Leng et al. [50] used BM-derived MSCs intravenously in 7 patients with COVID-19 (1 with critically severe illness, 4 with severe illnesses, and 2 with common illnesses). Mass cytometry and cytokine analysis of the patients’ peripheral blood also showed loss of over-activated T cells and NK cells, an increase in anti-inflammatory cytokines like IL-10, and a decrease in pro-inflammatory cytokines such as TNF-alpha. Hence, it shows that MSC has a potential to decrease cytokine storm.

Cell- and tissue-based remedies such as stem cell therapy, NK therapy, T-cell therapy, chimeric antigen receptor, stem cell-exosomes, extracellular vesicles, and tissue products are of considerable therapeutic interest against infectious diseases [151, 152]. Despite being at the initial stages, the use of MSCs against COVID-19 has attracted substantial attention as regenerative medicine. As stem cell treatments have already revealed positive outcomes in patients with pulmonary pathologies, there are prospects of hopeful therapies using assorted types of MSCs in immunomodulation, regenerative medicine, cell or tissue engineering, and anti-inflammatory treatments [89]. The clinical studies which ascertained the efficacy of stem cell therapy have been highlighted in Table 1.

**Table 1:** Summary of the clinical studies of stem cell therapy against COVID-19

Number of patients	Symptoms	Doses	Duration of patient observation	Outcomes	References
18	Moderate and severe pulmonary disease	3 × 10^7^ cells per infusion	06 days	Intravenous hUCMSC infusion declined interleukin (IL)-6 levels and found to be safe. Adverse effects like high fever were noticed.	[148]
01	Lung inflammation, fatigue, fever, cough	Three times hUCMSC (5 × 10^7^ cells each time)	04 days	Remission of the lung inflammation symptom. The studies show the safety of cell doses	[101, 142]
01	Severe shortness of breath, cough, chest tightness, and fever	1 × 10^6^ hWJCs cells per kilogram of weight	07 days	Effective against COVID-19 pneumonia	[84]
10	Respiratory distress, fever	1 × 10^6^ MSCs per kilogram of body weight	14 days	Reduction in peripheral lymphocytes, cytokine-secreting immune cells CXCR3 + CD4+ T cells, CXCR3 + CD8+ T cells, CXCR3 + NK cells disappeared in 3–6 days.	[50]
12	Fever, chest tightness, shortness of breath, and fatigue	2 × 10^6^ cells/kg	28 days	Intravenous infusion of hUCMSC reduced the lung inflammation, as well as interleukin (IL)-6 levels, ascertained as an effective option to cure severe COVID-19	[153]
13	COVID-associated pneumonia	0.98 × 10^6^ AT-MSC/kg	16 days	Decrease in inflammatory parameters (reduction in C-reactive protein, IL-6, ferritin, LDH, and d-dimer), as well as an increase in lymphocytes	[154]
02	Fever and dyspnea	1 × 10^6^ MSCs per kilogram of body weight	14 days	Lymphocytes increased, the inflammation mediators declined, symptom of dyspnea improved	[155]
24	Classic ARDS, chronic obstructive pulmonary disease	15 ml ExoFlo™ (derived from MSCs) + 100 ml normal saline	14 days	Increased lymphocyte and neutrophil count, reduction was noted in C-reactive protein, IL-6, and ferritin	[156]

In view of the importance of stem cell therapies in COVID-19, there is a need to generate patient-specific clinical-grade immunocompatible cells, ESCs and MSCs. We have emphasized SCNT cloning as a futuristic tool to generate stem cells from fetal, neonates, or adult human donors. The stem cells generated by SCNT and induced pluripotency are promising tools for personalized requirements of regenerative medicine, transplantation, and disease modeling [157].

## SCNT cloning for biomedical and regenerative medicine

**Fig. 1:**
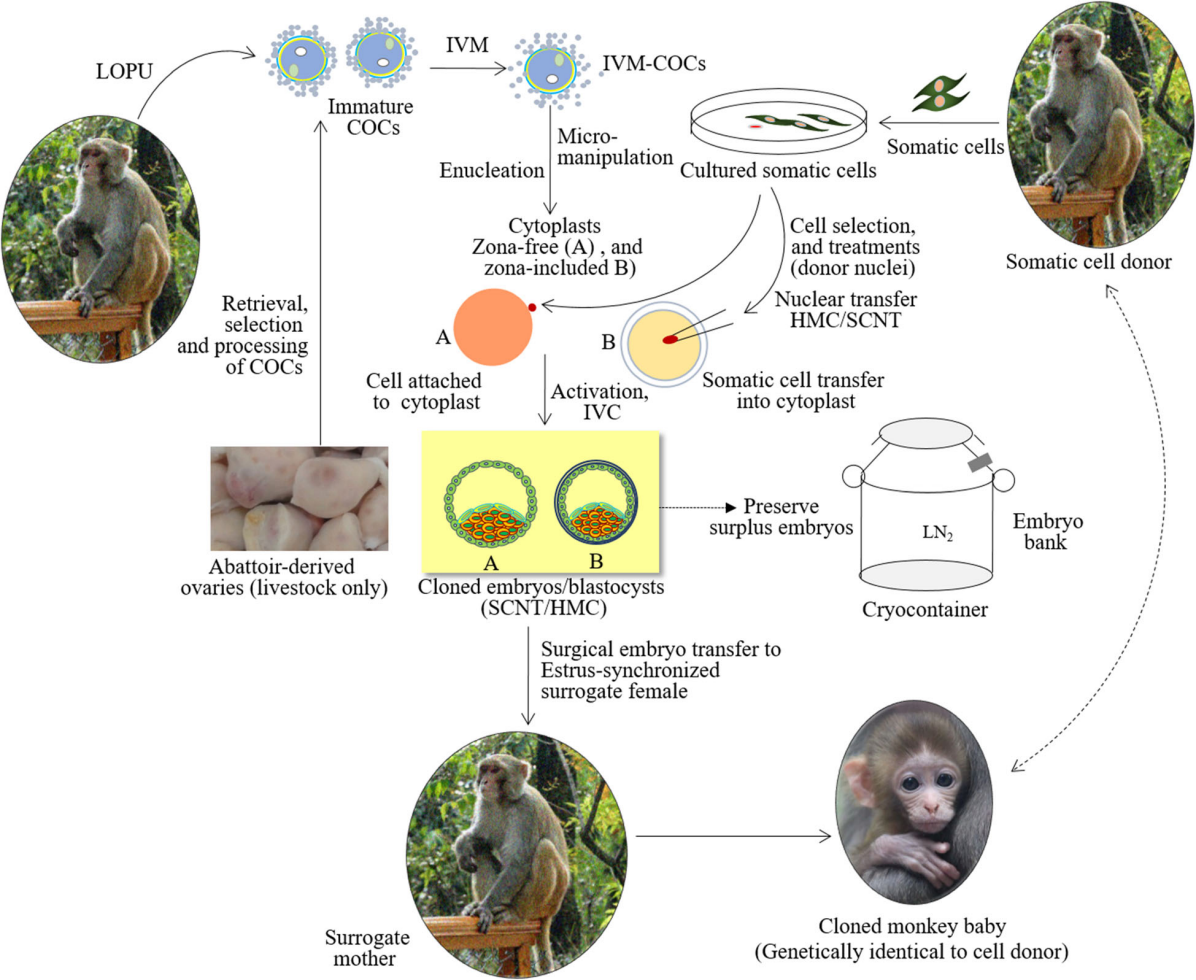
A simplified depiction of basic protocols of SCNT cloning showing monkey as NHP representative. SCNT pioneered a new era in stem cell engineering and cellular medicine by proving that it is possible to reprogram the genome of mature cells to a totipotent stage comparable to embryos. Here, COCs, cumulus oocytes complexes, i.e., immature oocytes; IVC, in vitro culture of cell-cytoplast couplets/embryos; IVM, in vitro maturation; HMC, handmade cloning; LOPU, laparoscopic ovum pick-up

SCNT initially introduced in 1952 to discover embryo development in frogs came into broad publicity in 1997 when Prof. Ian Wilmut and his team succeeded to reprogram the sheep fibroblasts and produced “Dolly,” the first cloned mammalian species [158]. The technique (Fig. 1) has been modified and adapted to clone several mammalian species, including laboratory animals, livestock, and endangered wild mammals including non-human primates (NHPs) [159]. The underlying principle of SCNT is that the body cells in an individual possess an identical genome despite being different in phenotypes, niches, and functions. SCNT organizes the reprogramming of the genome of donor nuclei and accordingly provides the means of transforming a matured cell to a totipotency state comparable to an embryo. Hence, SCNT cloning circumvents the processes that generally ensue during gametogenesis and fertilization and enable the embryo to undergo normal development.

Transgenic animal models are important bioresources for developmental biology and biomedical research. Highly exigent need of unraveling, preventing, and controlling the SARS-CoV-2 necessitates the search for optimal animal models. The paucity of a befitting COVID-19 preclinical model animal is one of the major impediments. The researchers are in race to understand the molecular mechanisms of SARS-CoV-2 infection to repurpose the currently available drugs to develop animal models and antiviral therapies against the pandemic. NT cloning is used to generate stem cells and progenies characteristically identical to the already borne live or deceased organism.

Coronaviruses affect a broad range of mammalian species. Wild-type or genetically modified rodents, pigs, and NHPs have been of paramount interest and provide key insights into the molecular basis of health and diseases. Notably, small animals such as hamsters, cats, ferrets, and NHPs are variably susceptible to SARS-CoV-2 infection [160]. These animals, however, are not suitable as models for SARS-CoV-2 pathogenesis and drugs and vaccine testing. On contrary, mouse (*Mus musculus*) is a well-studied laboratory animal in various pathogenesis studies and drug testing, unlike humans, the wild-type mouse strains are resistant to SARS-CoV-2. As mouse ACE-2 does not effectively bind to virus spike protein, the SARS-CoV-2 infects mice only when they are genetically modified to express human ACE2 [160].

Initially, laboratory small animals such as rodents (mice and rats) [161] and large animals such as pigs [162, 163] were cloned for biomedical studies. Primates serve as superlative models to study human-associated diseases such as cognitive functions and brain disorders. Healthy clones of macaque monkeys (*Macaca fascicularis*) have been produced [164]. SCNT cloning of NHPs combined with gene editing (e.g., CRISPR-Cas9) may begin a new era of human clinical sciences to decipher and cure human genetic diseases [164].

Genetically modified animals viz., rodents, pigs, and NHPs open biologically safe and time-saving avenues and provide important insights into the molecular basis of health and diseases. Among 14 animal species screened for their ACE-2 receptor homology to that of humans’ ACE-2 functional receptor, the rhesus macaques have shown to be the closest match [165].

Though various strategies such as sequential passaging of the virus in murine lung and intestinal tissue [166] and adaptation of SARS-CoV-2 by reverse genetics aimed to modify the receptor-binding domain of SARS-CoV-2 are proposed to enhance infectivity in mice [167], few rare viruses in the normal swarm possessing mutations in spike proteins are selected based on their high affinity to ACE2 and the subsequent higher rates of replication in murine pulmonary cells.

It can be envisaged that cells obtained from animals already expressing human ACE2 might serve as donors to propagate genetically modified cloned offspring, embryos, or NT-ESCs for use as models to investigate SARS-CoV-2.

## Generating cells for clinical applications

In biomedical sciences, the reprogramming refers to the erasure and remodeling of epigenetic genomic marks such as DNA methylation during mammalian development or cell culture. Genome reprogramming is a speedy and large-scale (10–100% of epigenetic marks) process which completes at three stages, namely gametogenesis (in primordial germ cells), fertilization of the ovum by sperm, and early embryonic stages.

Genome reprogramming is a highly complex and partly understood biological process. Partially activated G0/G1 arrested quiescent cell or G2 or M phase cell [168] is introduced into perivitelline space of the cytoplast (enucleated oocyte) followed by its electrofusion with recipient cytoplast. The ooplast reprograms the donor cell genome [169]. Quickly after the transfer of the somatic cell into the cytoplast, the maturation/meiosis/mitosis-promoting factor (MPF) [170] triggers nuclear membrane breakdown to produce a condensed-metaphase-like chromosome through a cascade mechanism collectively known as premature chromosome condensation (PCC). Notably, the PCC is important and determines the development of the reconstituted embryo. In natural fertilization, the sperm-specific phospholipase Cζ (PLCζ) acts as a mediator of oocyte activation, helps exit its M phase, and commences embryo development [171]. As PLCζ lacks in somatic cells, and no sperm interaction is involved in SCNT cloning, the reconstituted embryo essentially needs simulated activation to undergo auxiliary development. Various supplements, i.e., artificial oocyte activators (AOAs), such as calcium ionophores or SrCl_2_ incorporated into embryo culture medium, activate the reconstituted embryos.

Sperm and oocyte pronuclei are present in fertilized zygotes, whereas nuclei in reconstituted or SCNT embryos are called pseudonuclei. Subsequent events in the reprogramming and development include nuclear expansion of the pseudo-pronucleus, zygotic genome activation, and chromatin reprogramming including histone modification reprogramming, gene methylation reprogramming, and transcriptome reprogramming. Overall, the SCNT reprograms the epigenetic status of somatic cells used as donor cells within a brief period though few regions are not reprogrammed, or they resist reprogramming [169].

## Regenerative medicine

Inner cell mass (ICM) cells of cloned embryos serve as a source of NT-ESCs. In humans, nuclear transfer cloning is used to generate patient-specific NT-ESCs which are isogenic and immunocompatible. This process is known as therapeutic cloning. Handmade cloning (HMC) [172, 173], a modified procedure of nuclear transfer cloning, is simpler as it circumvents dependency on expensive micromanipulation to produce cytoplast from IVM oocytes and transfer of donor cell across zona pellucida of the cytoplast.

NT-ESCs can be propagated, preserved, and de-differentiated into other cell types. Notably, the mammalian cells can also be reprogrammed by ectopic or induced expression of exogenous genetic factors *Oct5*, *Sox2*, *Klf4*, and *cMyc* (OSKM), also known as Yamanaka factors [174]; non-genetic elements and small molecules [175]; microRNAs [176]; synthesized transcription factors [177]; combinations of chemical compounds [178, 179]; and cell fusion [180]. The phenomenon of intestine-specific caudal-related homeobox (CDX), especially the CDX1-induced SALL4 and KLF5-mediated intestinal epithelial cell reprogramming into tissue stem-like progenitor cells [181], has led to the concept of using beneficial microorganisms such as lactic acid bacteria to reprogram the somatic cells [182, 183]. In nuclear transfer cloning, the cytoplast possesses mitochondria and other factors that support the metabolism competency, cope with metabolic oxidative stress, and assist rejuvenation of donor cells [184].

## Organoids technology and COVID-19

Organoids are tiny, self-organized 3D tissue derived from adult cells, ESCs, or reprogrammed stem cells, i.e., iPSCs, which recapitulate much of the complexity, selected properties, and genetic signatures of original tissues and organ [185, 186]. Organoids bridge the preclinical and clinical science and have resolved various research anomalies and therapeutic challenges. Like different types of the organs, the organoids are also of different types and are analyzed by various sequencing methods, molecular imaging, and spectrometry. Multiple organ stem cell-derived organoids have been developed for disease modeling, host-pathogen interactions, drug discovery, regenerative medicine, and studying organogenesis.

Human organoids generated from patient biopsies or stem cells are used for drug screening and study biomedical complications, genetic disorders, infectious diseases, and disease modeling with high precision. Human iPSC-derived monolayer brain cells and region-specific brain organoids have revealed that compared to choroid plexus epithelial cells, the neurons and astrocytes are sparsely infected with SARS-CoV-2 [187]. Human stem cell lung organoids were found to be susceptible to SARS-CoV-2 infection exhibiting vigorous induction of chemokines [188]. Analysis of human airway organoids shows that SARS-CoV-2 has multi-basic cleavage sites in its spike protein which increase its infectivity towards airway cells, and compared to other coronaviruses, the SARS-CoV-2 enters more rapidly into airway cells [189]. High-throughput screening of interaction of the human lung organoid model with some FDA-approved drugs against COVID-19 has shown that imatinib, mycophenolic acid, and quinacrine dihydrochloride could significantly inhibit the SARS-CoV-2 [188]. Hence, organ-specific stem cell-derived organoids provide valuable tools to identify therapeutics and drugs against COVID-19. Stem cells generated by induced pluripotency or SCNT reprogramming might be a valuable resource to generate multiple types of organoids to screen efficacy and safety of drugs, basic virology, and pathogenesis of SARS-CoV-2.

## SCNT vis-à-vis bio-pharming

The transgenic cloned animals have several applications in research, medicine and agriculture. Recombinant proteins produced through transgenic animals have post-translational maturity and stability. Compared to cultured animal cells, transgenic mammalian species serve as an excellent platform to produce monoclonal antibodies (mAbs) in milk. An improved version of cetuximab, a mAb against epidermal growth factor receptor, is produced at a larger scale in transgenic goats [190].

Gene-edited animals that serve as donors of clinical-grade stem cells are produced by SCNT [191–193]. A line of transgenic goats has been designed to express human lysozyme in their mammary glands [194]. Targeted changes in the animal genome are likely to initiate a new era of bio-pharming [195]. Healthcare applications include production of target-specific stem cells and therapeutic proteins [196], mAbs [190], released into the milk of animals, to the use of genetically modified (GM) animals to produce organs for xenotransplantation are envisaged.

The use of cloned mammalian livestock (goats, pigs, and cattle) for commercial production of recombinant human proteins and nutraceuticals has been reviewed elsewhere [197–199]. Currently, information is lacking on the synthesis of recombinant SARS-CoV-2 S protein using cloned transgenic mice, rabbits, pigs, or milch animals such as goats, sheep, or cattle. More recently, recombinant SARS-CoV-2 S protein has been produced using baculovirus-silkworm expression system. S proteins secreted into silkworm serum have been purified and would be used for the development of immunodetection, immunoglobulin, and vaccine development against the virus [200]. Delay in development of cloned transgenic animals as model animals or source of recombinant SARS-CoV-2 proteins is probably due to low efficiency of SCNT cloning to produce embryos, long gestation periods, more age of achieving maturity, and expression of recombinant proteins in mammary tissue and excretion into milk.

## Development of model animals and therapeutic cells

There is an urgent need of animal models to screen and evaluate vaccines and drugs to treat COVID-19. SCNT is at present the most reliable method to produce cloned and transgenic animals including livestock. However, SARS-CoV-2 pathogenicity is studied using macaques, ferrets, cats, and hamsters. NHPs are instrumental for the preclinical evaluation of vaccines against COVID-19 [104]. Transgenic mice expressing human ACE2 are the currently in vivo systems to discover SARS-CoV-2 [201, 202].

**Fig. 2:**
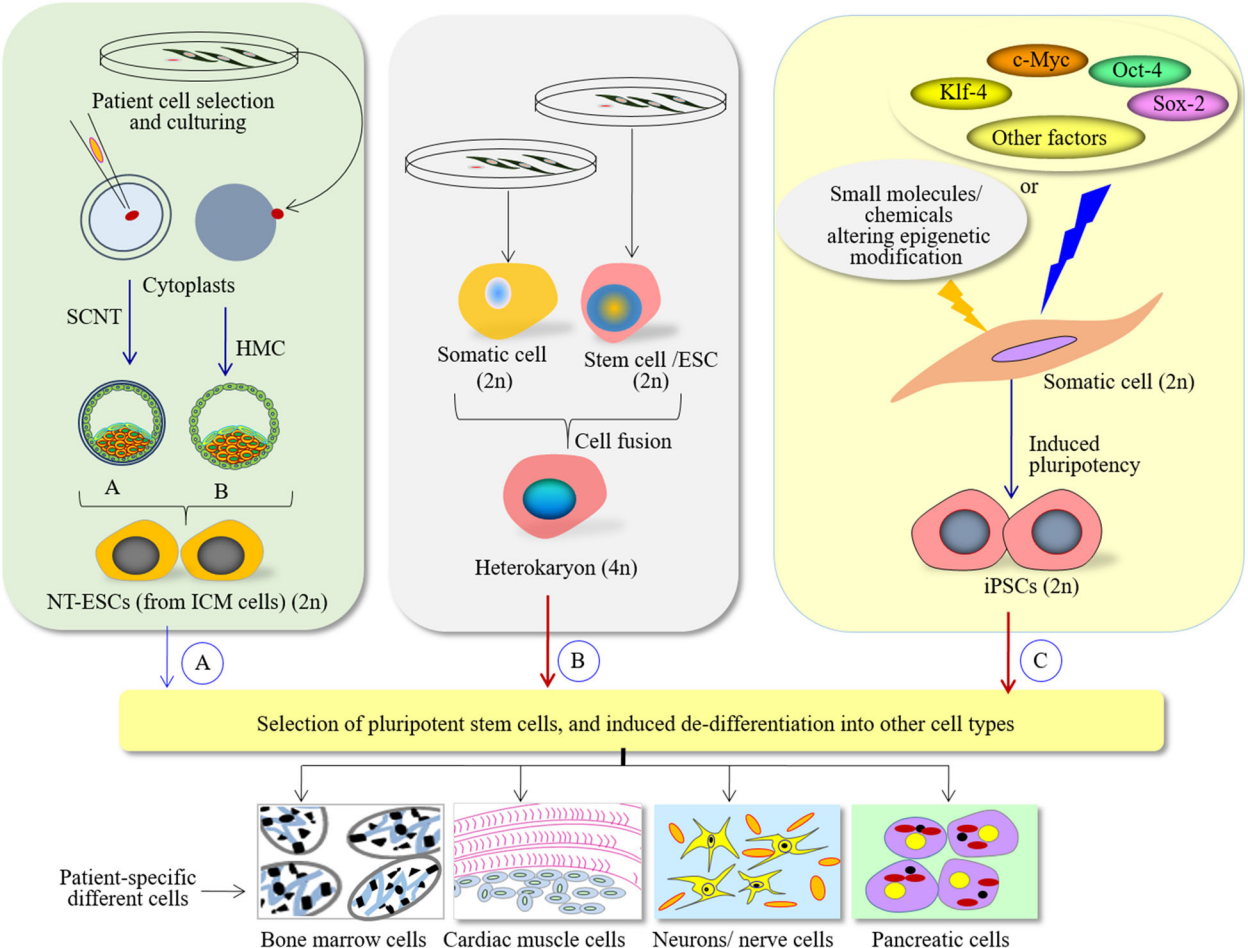
Various strategies: (a) SCNT reprogramming, (b) somatic cell fusion, and (c) induced pluripotency to reprogram patient-specific somatic cells to generate cells for regenerative medicine. Genetic methods viz., nucleic acids (NA), synthetic mRNA, non-nucleic methods (proteins and small molecules), and epigenetic modifiers such as vitamin C, 5-azacytidine, trichostatin A, valproic acid, sodium butyrate assist genome reprogramming. Compared to other methods, SCNT is a safe approach to generate NT-ESCs, which can be de-differentiated into other cell types

Though some vaccines are already underway, there is a need to discover alternative therapeutics to target SARS-CoV-2, or associated health complications, such as dysregulated immune responses and systemic problem arising from COVID-19 [201]. There is a surge in the demand of patient-specific isogenic cells for drug testing. Stem cells offer opportunities to advance the cellular medicine due to their ability to de-differentiate into any type of body cells [203, 204]. Therapeutic cloning of somatic cells to a pluripotent NT-ESC state is used to create multiple histocompatible cell types, thus overcoming the possibilities of immune rejection of the transplanted tissues [205]. Compared to other methods of genome reprogramming (Fig. 2), SCNT reprogramming of cells is anticipated to generate patient-specific therapeutic grade cells. The iPSCs generated by OSKM have repulsive concerns that restrict their use in biomedical applications. The use of retroviruses may cause cancer or tumor formation. In addition, retroviruses insert their DNA into the host genome and trigger the expression of cancer-causing genes. *C-Myc* (one of the genes used in reprogramming) is a known oncogene whose overexpression can induce teratoma. Moreover, all the pluripotency factors are not equally expressed, and reprogramming of non-dividing cells such as peripheral blood mononuclear cells (PBMC) and aged skin fibroblasts is very low. Nonetheless, the retroviral vectors, epiosmal vectors, and Sendai viruses had a comparable reprogramming efficiency and did not affect gene expression in fibroblast-derived human iPSCs [206].

**Fig. 3:**
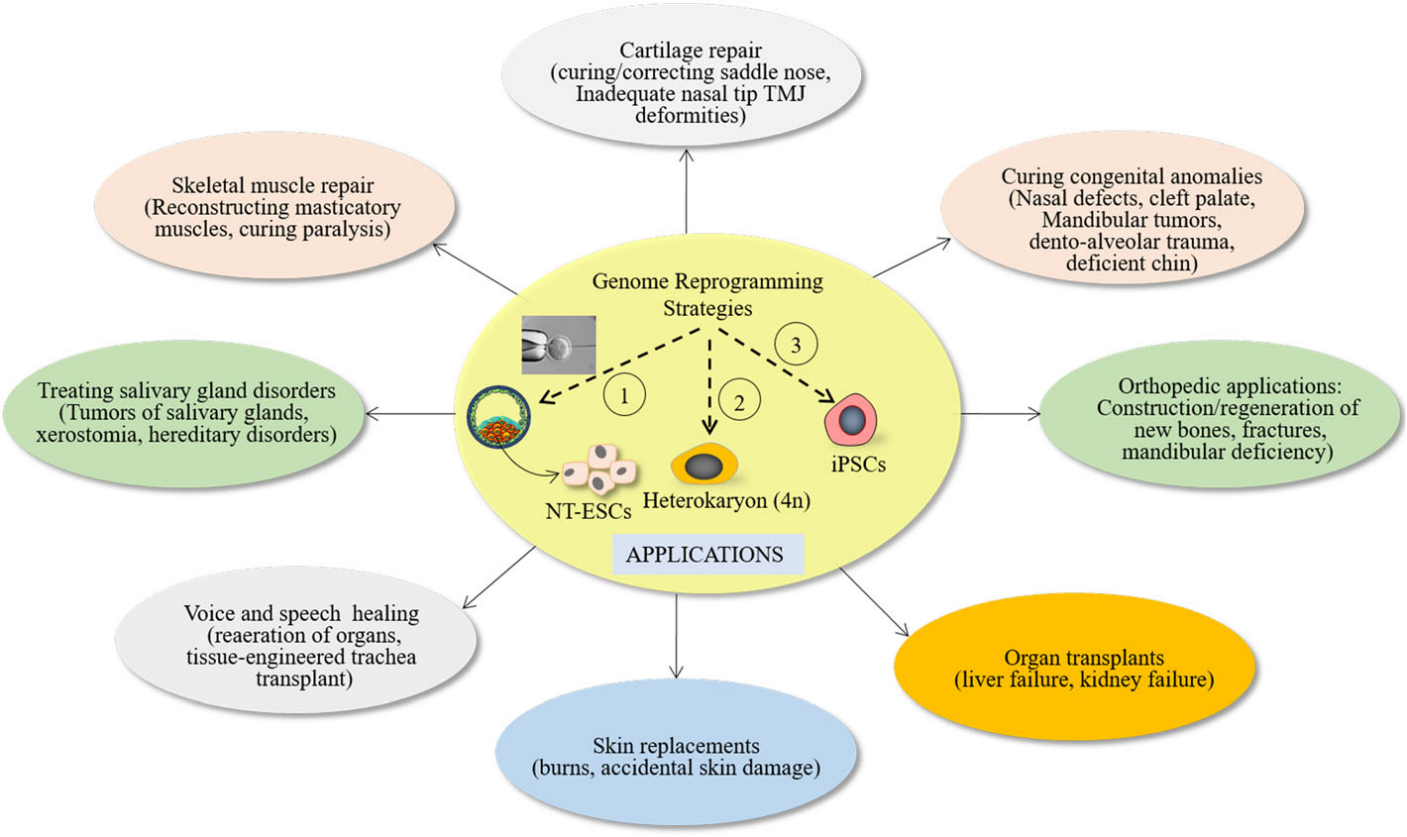
Salient applications of reprogrammed cells in regenerative medicine, bio-pharming, and biomedical sciences. Human stem cell-derived organoids are important for testing host-virus interaction and screening efficacy of drug molecules against COVID-19. Here, 1, SCNT; 2, cell fusion; 3, induced pluripotency to generate iPSCs

Patient-specific NT-ESCs serve as valuable in vitro disease modeling and drug screening tools. NT-ESCs contain mitochondrial DNA (mtDNA) exclusively from the ovum or oocyte. Consequently, NT-ESCs generate tissues that are metabolically active or functional and are apt for cell therapies irrespective of mtDNA of donor cells. Hence, SCNT is a desirable strategy for accurate mtDNA mutations and rescuing stem cells’ metabolic function in patients with mtDNA abnormalities [207]. The remarkable ability of ESCs to transform into any other type of cell has allowed rapid progress towards the treatment of SCI, hemophilia, and multiple sclerosis. Figure 3 summarizes salient applications of SCNT and NT-ESCs in regenerative medicine and human biomedical sciences.

Development of patient-specific stem cells from SCNT embryos or iPSCs is a fundamental requirement in regenerative medicine to treat the diseases with minimal probabilities of immunorejection. So far, most regenerative cells reported are from small animals such as rodents. As compared to rodents, NHPs share more homology with human physiology, genomics, immune system, and the basic biochemistry; NHP stem cells may serve to study human diseases authentically.

Using *Chlorocebus sabaeus* monkey oocytes and somatic cells, Chung et al. [191] have developed NT-ESCs and iPSC lines to study neurological disorders such as Parkinsonism. The NT-ESCs expressed stemness markers and differentiated into all the cells representatives of three germ layers [191].

## Future directions, outlook, and challenges

Genome reprogramming which enables transformation of differentiated or matured cells into an undifferentiated totipotent cell state is highly useful techniques to obtain isogenic immunocompatible cells for therapeutic applications. Among different methods of reprogramming mammalian cells, nuclear transfer cloning provides novel opportunities to stem cell engineering, developing organoids besides its use in producing cloned animals for recombinant proteins, nutraceuticals, and tissues.

The discovery of regenerative medicine is at par with common vaccinology. In this perspective, SCNT is an exquisite tool in biomedical and veterinary health. However, the trickiest challenges are its low efficiency and developmental abnormalities resulting due to imperfect epigenetic reprogramming.

Poor-quality pre-implantation embryos or blastocysts resulting due to incomplete reprogramming or aberrant development such as delayed development, low number of blastomeres or embryo cells, and genome instability impede yield of ESCs. Another issue crucial for the success and implementation of SCNT in humans and primates is the availability of oocytes for research purposes. It evidently involves financial and ethical implications. It is envisaged that research on applications of SCNT and the iPSCs to produce sperm and oocytes is of high biomedical importance and will continue in near future [208].

The fundamental hurdles related to animal cloning need to be resolved by analyzing underlying cellular and molecular mechanisms. Mitochondrial proteins (Mfn2 and Bcnl3L) deregulation or dysfunction in cloned embryos hampers their development [209]; hence, multiple strategies should be developed to overcome aberrations in mitochondrial protein expression.

Nuclear transfer cloning is a persuasive research tool to generate distinctive cell type or 3D tissue models (organoids) for disease pathogenesis and regenerative medicine. In particular, patient-specific NT-ESC-derived lung organoids can serve as a disease model to study SARS-CoV-2 infection and drug screening tools to identify candidate COVID-19 therapeutics. There is a need to develop an analysis of brain-, lung- and kidney cell-derived organoids and study them to determine differential organ-specific SARS-CoV-2 tropisms. This will help to determine infection susceptibility of different cells, mechanisms of SARS-CoV-2-induced cell dysfunction, and treatments apart from reducing the need for animal experiments.

Concomitant use of human iPSCs and CRISPR/Cas9 has novel prospects and opportunities to drug screening and developing biotherapeutics to prevent infectious and non-infectious diseases. SCNT technology could add to modern medicine when pooled with CRISPR/Cas9-mediated genome editing. As potent genome altering tools, iPSCs and CRISPR/Cas9 have advanced basic and translational research and allow deep insights into developmental biology and pharmaceutical research [210, 211].

One distinctive feature of SCNT is that it enables the direct generation of embryo and organisms from single cultured or genetically modified donor cell. Further, NT cloning is a desirable method of generating stem cells without tumorigenic factors such as *cMyc* as genome-reprogramming factor, and early embryo development are elicited by ooplast components, electric impulse, and chemicals as supplements in pre-implantation stages of embryo culture media. This feature allows opportunities of rapid and efficient generation of cloned CRISPR/Cas9 genome-edited experimental animal models.

Xenotransplantation of organs is a promising strategy to alleviate the shortage of organs for humans. Additionally, in combination with gene editors such as CRISPR-Cas9, SCNT can rapidly produce gene-edited cloned animals such as pigs and livestock with desirable traits such as fast growth rate, resistance to biotic and abiotic stress, and short breeding interval. It is need of the hour to produce an animal model expressing human ACE2 to study the pathogenicity of SARS-CoV-2 and gene-edited animals to fulfill the demand for therapeutic proteins, immunocompatible cells, tissues, and organs for human transplantation [212, 213].

Moreover, MSCs, NT-ESCs, cells and tissues, and organoids will advance the understanding of the pathophysiology of COVID-19, can contribute to study SARS-CoV-2, and adapted to assess potential drugs or vaccines. Hence, advancements in SCNT will further benefit human regenerative therapies by propagating transgenic and genetically modified animals to study SARS-CoV-2 and its interaction with host.

In conclusion, COVID-19 caused by SARS-CoV-2 and its emerging variants is a serious pandemic and necessitates multiple level interventions to stop health problems. Therapeutic cloning in its present embodiment is a viable alternative to develop ESCs, cells, and tissues from an individual of any age. SCNT is a widely used technique to clone early-stage embryos and obtain stem cells, stem cell-derived gametes, organoids, exosomes, and transgenic animal models for biomedical applications. The efficiency of SCNT needs improvements to exploit its copious potential in biomedical sciences.

## Data Availability

Not applicable.

## Abbreviations

SCNT: Somatic cell nuclear transfer
MSCs: Mesenchymal stem cells
ARDS: Acute respiratory distress syndrome
ESCs: Embryonic stem cells
NT-ESCs: Nuclear-transfer embryonic stem cells
COVID-19: Coronavirus disease 2019
SARS-CoV-2: Severe acute respiratory syndrome coronavirus 2
SCI: Spinal cord injuries
EPCs: Endothelial progenitor cells
iPSCs: Induced pluripotent stem cells
NHPs: Non-human primates
IL: Interleukin
NK: Natural killer
TLRs: Toll-like receptors
TNF: Tumor necrosis factor
hUCMSC: Human umbilical cord MSC
EVs: Extracellular vesicles
MPF: Mitosis-promoting factor
PCC: Premature chromosome condensation
HMC: Handmade cloning
mAbs: Monoclonal antibodies
GM: Genetically modified
NA: Nucleic acids
mtDNA: Mitochondrial DNA
PBMC: Peripheral blood mononuclear cells
